# Synergistic self-driven and heterogeneous effect of a biomass-derived urchin-like Mn_3_O_4_/C_3_N_4_ Janus micromotor catalyst for efficient degradation of carbamazepine†

**DOI:** 10.1039/d4ra04980b

**Published:** 2024-09-12

**Authors:** Jie Yang, Wenning Yang, Chao Zhang, Jian Gong, Ming Xu, Jia Li, Chengzhang Liu

**Affiliations:** a Department of Pharmaceutical and Bioengineering, Zibo Vocational Institute Zibo 255000 P. R. China; b Shandong Provincial Key Laboratory of Chemical Energy Storage and Novel Cell Technology, School of Chemistry and Chemical Engineering, Liaocheng University Liaocheng 252000 P. R. China; c School of Artificial Intelligence and Big Data, ZiBo Vocational Institute Zibo 255000 P. R. China 652143914@qq.com; d State Key Laboratory of Chemical Resource Engineering, Beijing University of Chemical Technology Beijing 100029 P. R. China; e School of Material Science and Engineering, University of Jinan Jinan 250022 China mse_lij@ujn.edu.cn

## Abstract

It is well known that obtaining efficient carbamazepine degradation materials or rapid carbamazepine-removal methods is still a challenge in the field of environmental remediation. Hence, the present study aimed to concurrently address these issues by combining a self-driven, heterostructured and low-cost biomass-templated urchin-like Janus micromotor catalyst for highly efficient carbamazepine degradation. The catalyst could autonomously move in a circle-like motion pattern *via* O_2_ bubbles generated from the Mn_3_O_4_-catalyzed decomposition of H_2_O_2_ with a velocity of 223.5 ± 7.0 μm s^−1^ in 1% H_2_O_2_. Benefiting from the well-structured heterojunction at the interface of C_3_N_4_ and Mn_3_O_4_, carbamazepine (CBZ) was degraded by 61% in 100 min under sunlight irradiation. In addition, density functional theory calculation results proved that the formation of the heterojunction structure promoted the generation of photo-generated carriers. Thus, the presented method provides a promising pathway for the rational construction and preparation of movable catalysts for the efficient removal of organic pollutants from wastewater.

## Abstract 2

Here, we present biomass-derived urchin-like Mn_3_O_4_/C_3_N_4_ Janus micromotor catalyst for efficient degradation of carbamazepine. This method provides a promising pathway for rational construction and preparation of movable catalysts for efficient removal of organic pollutants from wastewater.

## Graphical abstract

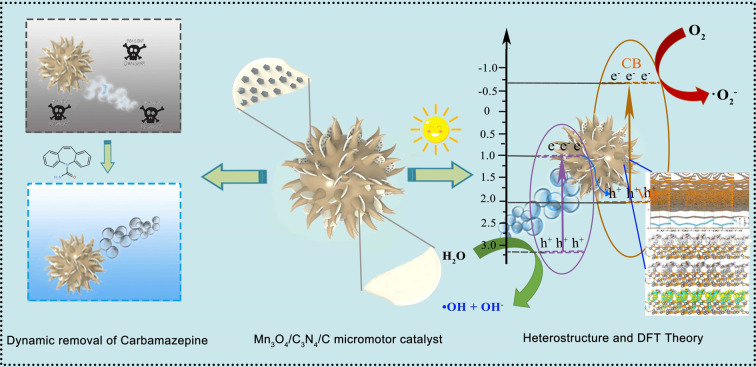

## Introduction

Antibiotic degradation remains a great challenge to green, low-cost, and feasible approaches for the degradation of medicines and environmental protection. As one of the broad-spectrum antibiotics, carbamazepine (CBZ) has attracted much attention in the treatment of various diseases such as trigeminal neuralgia, epilepsy, and mental illness.^1^ According to statistics, about 28% of CBZ is discharged into the water environment due to incomplete human metabolism and medical sewage discharge.^2^ To worsen the situation, CBZ is a kind of extremely durable and stable organic environment pollutant and cannot be effectively removed *via* traditional wastewater treatment technologies.^3,4^ Therefore, the development of efficient treatment technology for the degradation of CBZ in sewage is urgent. Compared with the removal of CBZ using conventional technologies, such as biological treatment, adsorption, and other methods,^5–7^ the advanced oxidation process (AOP) is regarded as a promising method for the effective removal and mineralization of CBZ.^8–10^ Specifically, AOPs based on heterojunction photocatalysis have received more attention owing to their economical preparation, convenient and simple synthesis, and efficient degradation.^11–13^ Heterojunction structures have excellent electronic structural characteristics and can exhibit higher catalytic efficiency than traditional catalysts in catalytic reactions. Moreover, the large specific surface area and nanoparticle structure of heterojunction catalysts make their catalytic performance more stable than traditional catalysts. Further, their interactions and interface effects during the reaction process help prevent catalyst failure and deactivation.^14,15^ Among the many heterojunction photocatalysts, Mn_*x*_O_*y*_/C_3_N_4_, especially two-dimensional (2D) nanostructured Mn_3_O_4_ heterojunctions, has attracted great interest because of its delocalized conjugative structures, efficient charge separation, outstanding chemical stability, and low cost.^16–18^ It has also been reported that Mn_*x*_O_*y*_/C_3_N_4_ heterojunctions have good optical absorption properties and can be used for direct charge-transfer collection and are thus applied in a wide range of fields.^19–21^ Shi successfully prepared a g-C_3_N_4_/α-MnO_2_ Z-scheme heterojunction, which showed excellent visible-light photocatalytic performance, superior to its pure constituent parts.^22^ Moreover, Zhang reported a porous MnO_2_/Mn-modified alkalinized g-C_3_N_4_ catalyst, which exhibited high catalytic activity and 96.7% tetracycline removal.^23^ Besides, Chen synthesized nanodot–nanosheet (Mn_3_O_4_/g-C_3_N_4_) composites, which showed the best performance in persulfuric salt (PMS) activation for the removal of 4-chlorophenol (4-CP).^24^ However, it is still a challenge to prepare efficient Mn_*x*_O_*y*_/C_3_N_4_ heterojunction composites to achieve efficient photocatalytic effects and describe the detailed mechanism of action of heterojunction structures.

At the same time, the satisfactory degradation performance of a catalyst depends not only on its inherent properties but also on the chance of it contacting with the target pollutants. In order to achieve more effective contact, making the catalyst disarray move is a very useful method to ensure achieving effective contact.^25–27^ In this regard, micro-/nanomotors, a kind of self-propelled device at the micrometre or nanometre level, that can convert different forms of energy into kinetic energy to perform special tasks under liquid conditions, have considerable application prospects in the fields of biomedicine, sensing, and environmental remediation. Compared with the traditional static catalytic degradation, micro-/nanomotors can shorten the reaction time and reaction site by increasing the contact frequency and changing the displacement of molecules.^28–31^ For instance, Zhu introduced a bioinspired flower-shaped hierarchical Pt-free micromotor with a maximal adsorption capacity of 129.51 mg g^−1^ through the assistance of movement.^32^ Song summarized methods utilizing micro-/nanomotors for improving biosensing, such as the sensitivity, selectivity, detection time, biocompatibility, simplified system operation, and environmental availability.^33^ Our group recently constructed a novel glucose-driven catalytic nanomotor with robust dual enzyme-like activities for the sensitive colorimetric sensing of glutathione (GSH) in wastewater.^34^ Therefore, it has been confirmed that self-propelled motion and bubble formation together lead to more effective fluid mixing, thereby increasing the degradation efficiency of low-concentration pollutants and compensating for the low diffusion rate of heterogeneous sensors and catalysts.

**Scheme 1:**
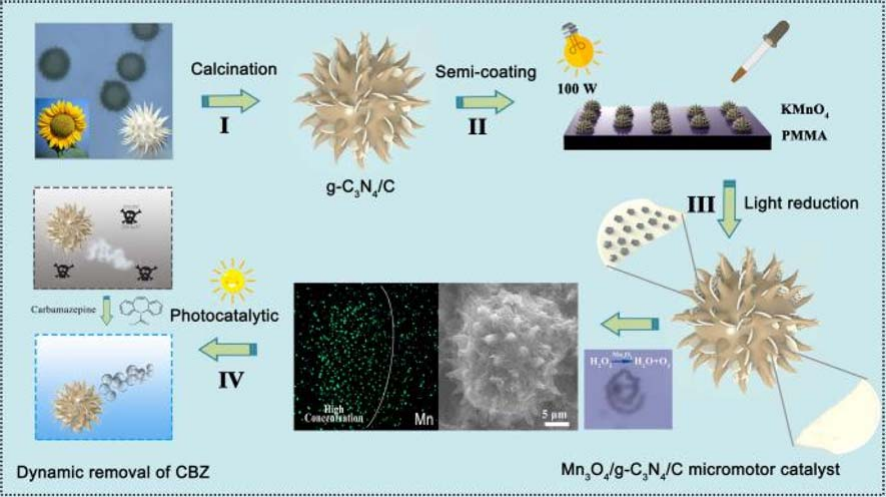
Schematic of the synthesis and application of a Mn_3_O_4_/C_3_N_4_ micromotor catalyst (Mn_3_O_4_/C_3_N_4_-JMC). (I) Growth of C_3_N_4_ nanosheets on sunflower pollens *via* calcination in an inert atmosphere. (II) Application of the organic solvent impregnation semi-coating method for catalyst preparation; (III) formation of Mn_3_O_4_/C_3_N_4_-JMC *via* a light-driven reduction process. (IV) Degradation of carbamazepine (CBZ) *via* the synergy of photocatalysis with autonomous motion.

Inspired by the previous biotemplate self-driven catalytic micro-/nanomotors and Mn_*x*_O_*y*_/C_3_N_4_ heterojunction composites, herein we synthesized a novel Mn_3_O_4_/C_3_N_4_ Janus micromotor catalyst (Mn_3_O_4_/C_3_N_4_-JMC) with a high specific surface area and 3D hierarchical structure, and applied this to the dynamic photocatalytic degradation of carbamazepine in the presence of hydrogen peroxide (Scheme 1). Sea urchin-shaped sunflower pollen with a grain size of 30 μm was soaked in hot phosphoric acid for several hours, and then washed several times with DI water, acetone, hydrochloric acid, and ethanol in sequence to remove sticky organic matter on the surface of the pollen. Next, a certain amount of melamine was thoroughly mixed and ground with the cleaned pollen. Then the mixture was calcined at 650 °C for 2 h under the protection of Ar gas in a tube furnace to obtain C_3_N_4_/C samples. Next, a certain concentration solution of PMMA was coated on one side of a glass slide. Then a small pinch of the C_3_N_4_/C samples was slowly poured onto the surface of the glass slide until PMMA evaporated to form a uniform thin film layer. Then, alkaline KMnO_4_ was dropped onto the slide glass. After that, the resulting hybrid composites were exposed to 100 W incandescent light for 12 h. Finally, Mn_3_O_4_/C_3_N_4_-JMC was obtained after a simple cleaning and drying process. In the presence of H_2_O_2_, on the one hand, the semi-coated Mn_3_O_4_ on the surface of the catalyst could decompose H_2_O_2_ to produce oxygen gas bubbles for propulsion. Meanwhile, heterojunction g-C_3_N_4_/Mn_3_O_4_ was able to degrade harmful carbamazepine in sewage under light irradiation. Benefiting from the synergistic effect of autonomous motion with the high efficiency catalysis of the Mn_3_O_4_/C_3_N_4_ heterojunction, the catalyst could highly efficiently degrade CBZ in water. Moreover, the catalyst system also demonstrated high reusability and stability. Therefore a new vision has been developed that has application potential in the field of environmental treatment and remediation.

## Experimental

### Chemicals and materials

Sunflower pollen was obtained from Taian Jinzhong Sanitary Material Co., Ltd. Melamine was obtained from Sinopharm Chemical Reagent Co., Ltd. Sodium dodecyl sulfonate (C_12_H_25_SO_3_Na, SDS), isopropanol (IPA), benzoquinone (BQ), sodium oxalate (OA), and phosphoric acid (H_3_PO_4_) were obtained from Tianjin Chemical Reagent Co., Ltd. Potassium permanganate (KMnO_4_), ethanol, acetone, hydrochloric acid (HCl), sodium hydroxide (NaOH), and hydrogen peroxide (H_2_O_2_) were purchased from Aladdin. Carbamazepine (CBZ), polymethyl methacrylate (PMMA), and ethyl acetate were purchased from Sigma-Aldrich. All the chemicals were of analytical-grade purity and were used as received without further purification.

### Characterization

X-Ray diffraction patterns were obtained using an X-ray diffractometer (Bruker D8-Advance, Germany, Cu X-ray sources, 40 kV), in the 2*θ* range from 5–80° and scanning speed of 2° min^−1^. The morphologies were analyzed by transmission electron microscopy (TEM) on a Tecnai F20 instrument at an accelerating voltage of 4 kV for the electron beam and by field emission scanning electron microscopy (FESEM) on a Quanta 400F system fitted with an energy-dispersive X-ray spectroscopy (EDX) unit. The concentration of the suspended sample was 0.2 mmol L^−1^. Fourier-transform infrared (FT-IR) spectra of the obtained samples were obtained using a Thomas Nicolet FT-IR spectrometer. The analyses were performed through KBr pellets and the wavenumber scanning range was 4000 to 400 cm^−1^, while the amount of samples was 20 mg, the spectral resolution was better than 0.4 cm^−1^, and the signal to noise ratio was 60 000 : 1. Simultaneous thermogravimetry and derivative thermogravimetry analyses (TGA/DTG) were carried out between 30 °C and 850 °C at a rate of 10 °C min^−1^ on a TA instrument (Netzsch Sta 449) under a flow of air. The surface elemental composition of the samples was determined by X-ray photoelectron spectroscopy (XPS). Videos of the Mn_3_O_4_/C_3_N_4_-JMC were captured by an optical microscope (Microscope N-300 M), coupled with a digital camera (Tucsen EC300) using the TSview software to analyze the speeds of the Janus micromotor catalyst. The photoelectric current was analyzed using an electrochemical workstation (CHI-600e) coupled with a standard three-electrode, where the sample-coated ITO glass served as the working electrode, Pt wire as the counter electrode, Ag/AgCl as the reference electrode, 0.1 M Na_2_SO_4_ solution as the electrolyte, and a 300 W Xe lamp as the radiation source.

### Synthesis of urchin-like C_3_N_4_/C

In the synthesis, the sunflower pollen grains were suspended in phosphoric acid and mixed to form a homogeneous suspension, which was further heated to 70 °C and stirred gently for 5 h. Then, the pollen grains were collected and extensively washed using DI water, acetone, hydrochloric acid, and ethanol in sequence. Finally, the wet pollen grains were dried at 60 °C for future characterization and experiments. Next, 0.5 g of the extracted samples and 3.0 g melamine were placed in a mortar and thoroughly ground for 5 min. Then the mixed samples were amplified in a tube furnace, under Ar protection at a heating rate of 2 °C min^−1^ for 4 h reaction at 650 °C. The obtained sample was designated as C_3_N_4_/C.

### Synthesis of the urchin-like Mn_3_O_4_/C_3_N_4_ Janus micromotor catalyst (Mn_3_O_4_/C_3_N_4_-JMC)

The Mn_3_O_4_/C_3_N_4_-JMC was prepared by a facile semi-coating method, as reported by Tan's research group.^35^ First, 50 mL of 10% w/w solution of PMMA was coated on one side of a 2.5 cm × 7.6 cm × 0.1 cm glass slide. Next, 0.05 g of the C_3_N_4_/C samples was slowly poured onto the surface of the PMMA-coated glass slide until the particles layer evenly covered the PMMA surface. Them, 0.1 mL of 0.45 M alkaline KMnO_4_ (KMnO_4_ : NaOH¼ 1 : 1 in molar ratio) was dropped onto the glass slide containing the C_3_N_4_/C. After that, the resulting hybrid composites were exposed to 100 W incandescent light for 12 h. Then the samples were cleaned with ethyl acetate and DI water followed by drying at 60 °C for 8 h, and the obtained products were denoted as Mn_3_O_4_/C_3_N_4_-JMC. Also, a comparative sample, Mn_3_O_4_/C_3_N_4_ without carbonized pollen, was prepared using an identical process.

### Removal of CBZ by Mn_3_O_4_/C_3_N_4_-JMC

The catalytic property of the as-synthesized products was reflected by CBZ removal under 300 W Xe lamp irradiation without sodium dodecyl sulfonate (SDS). In each experiment, 0.05 g of Mn_3_O_4_/C_3_N_4_-JMC samples was dispersed in a breaker containing 50 mL CBZ solution (50 mg L^−1^). This was then stirred in the dark for 30 min to achieve adsorption–desorption equilibrium and exposed to a xenon lamp at 30 ± 2 °C. Then a quantity of H_2_O_2_ (the total mass fraction, 0.5%) was added to the mixed solution. About 3 mL of the aliquot solution was withdrawn at different time intervals (0, 1, 3, 5, 10, 15, 20, 40, 50, 60, 100, 150, 200 min, *etc.*) from the reaction mixture, and was subjected to UV-vis spectrophotometry analysis. The absorbance at 284 nm was tested and recorded. The degradation efficiency of CBZ was calculated using the equation.

1 *D*(%) = (*C*_0_ − *C*_*t*_)/*C*_0_ × 100%

where *D* is the degradation efficiency of CBZ, *C*_0_ is the initial concentration, and *C*_*t*_ is the concentration after *t* min reaction of the solution.

### Stability and reusability of Mn_3_O_4_/C_3_N_4_-JMC micromotor catalyst

The operational reusability of Mn_3_O_4_/C_3_N_4_-JMC was evaluated in a series of repeated batch experiments, and the activity retention of the Mn_3_O_4_/C_3_N_4_-JMC was tested, as described in relation to the activity assays. After each batch, Mn_3_O_4_/C_3_N_4_-JMC was collected and washed with deionized water three times to remove any residual substrate and then reintroduced into the fresh reaction medium.

### DFT calculations

Density functional theory (DFT) calculations were performed using the Vienna *Ab initio* Simulation Package (VASP) with the projector augmented wave pseudopotentials.^36^ The Perdew–Burke–Ernzerhof (PBE) exchange–correlation functional within the generalized gradient approximation (GGA) was chosen in consideration of a balance between the accuracy and computational cost.^37^ The plane wave energy cut-off was 400 eV for the slabs. These periodic slabs were separated by 20 Å vacuum space along the *z* direction to isolate interactions between replicas. The electronic and force convergence standards were respectively set to 10^−8^ eV and 0.02 eV Å^−1^. The Brillouin zone was sampled on a 5 × 5 × 5 Monkhorst–Pack *k*-point grid for Mn_3_O_4_ bulk and 4 × 4 × 1 for the heterostructure and g-C_3_N_4_.^38^ The charge density difference was obtained by subtraction of that for the total heterostructure of g-C_3_N_4_/Mn_3_O_4_ minus that for g-C_3_N_4_ and Mn_3_O_4_. The latter two models' schemes did not undergo optimization.

## Results and discussion

### Characterization of the Mn_3_O_4_/C_3_N_4_-JMC micromotor catalyst

**Fig. 1:**
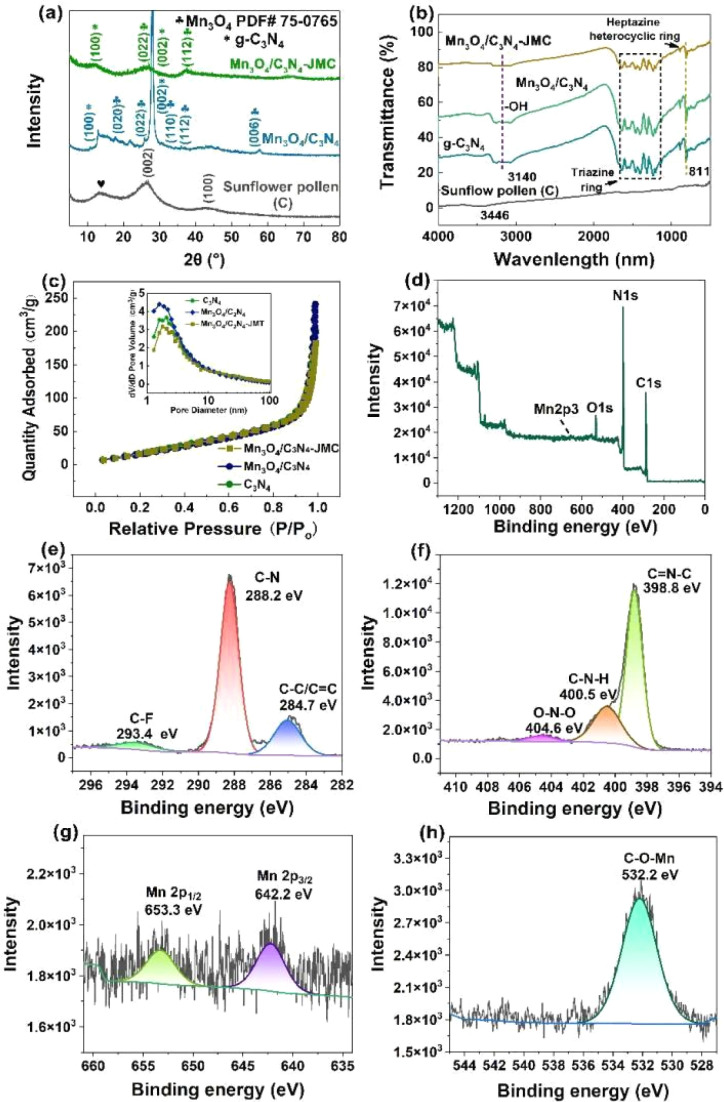
XRD patterns (a) and IR spectra (b) of the obtained samples; N_2_ adsorption–desorption isotherms and pore-size distributions of the obtained samples (c). Full-XPS spectra (d) and spectra of C 1s (e), N 1s (f), Mn 2p (g), and O 1s (h) for Mn_3_O_4_/C_3_N_4_-JMC.

Fig. 1a depicts the XRD patterns of the carbonized sunflower pollen and the final product Mn_3_O_4_/C_3_N_4_-JMC. The XRD pattern of a control sample Mn_3_O_4_/C_3_N_4_ is also provided for comparison. As shown, the carbonized sunflower pollen exhibited a high diffraction baseline and two broad diffraction peaks around 26° and 43°, ascribed to the (002) and (100) planes, indicating the presence of amorphous organics. Nevertheless, the presence of a broad diffraction peak at 13° marked by a symbol of spades could be ascribed to the graphite phase. In the XRD patterns of Mn_3_O_4_/C_3_N_4_ and Mn_3_O_4_/C_3_N_4_-JMC, the corresponding (100) facet at 12.81° indicated the periodic arrangement in triazine of C_3_N_4_; while the corresponding (002) crystal plane at 27.91° indicated the accumulation by the conjugated direction system of carbon nitride. Besides, the diffraction peaks at 17.84°, 25.87°, 30.61°, and 36.05°could be ascribed to the (020), (022), (110), and (112) planes of Mn_3_O_4_ (JCPDS 75-0765). At the same time, a low diffraction intensity was observed for the catalyst due to the large amount of amorphous carbon in the samples. Fig. 1b shows the FT-IR spectra of carbonized sunflower pollen, C_3_N_4_, Mn_3_O_4_/C_3_N_4_, and Mn_3_O_4_/C_3_N_4_-JMC. The peaks at approximately 811 cm^−1^ were mainly caused by the stretching vibration of the triazine ring in the graphite carbon nitride,^39^ while many peaks in the range of 1200–1700 cm^−1^ were attributed to the typical breathing and stretching vibration modes of the heptazine heterocyclic ring,^40^ and the peak at 3140 cm^−1^ corresponded to the –OH bond of water. Besides, the spectrum of Mn_3_O_4_/C_3_N_4_-JMC still appeared to be the same as the spectrum of C_3_N_4_, indicating the phase stability of C_3_N_4_ in Mn_3_O_4_/C_3_N_4_-JMC.

The nitrogen adsorption–desorption isotherms and pore-size distributions of C_3_N_4_, Mn_3_O_4_/C_3_N_4_, and Mn_3_O_4_/C_3_N_4_-JMC are shown in Fig. 1c. The isotherms of all these samples belonged to type IV isotherms with an H3 hysteresis loop, indicating the presence of mesopores in the obtained samples.^41^ Through calculation and analysis, the specific surface areas of C_3_N_4_, Mn_3_O_4_/C_3_N_4_, and Mn_3_O_4_/C_3_N_4_-JMC were 88.39, 89.66, and 81.35 m^2^ g^−1^, respectively (Table S1†). In addition, the average pore sizes of C_3_N_4_, Mn_3_O_4_/C_3_N_4_, and Mn_3_O_4_/C_3_N_4_-JMC were 7.1, 6.3, and 9.2 nm, respectively. As shown in the inset of Fig. 1c, the distribution of pores was mainly concentrated in the range of 2–50 nm and multi-peaks were observed for all three samples.

The elements C, N, Mn, and O could be clearly detected in the XPS spectra of Mn_3_O_4_/C_3_N_4_-JMC, as shown in Fig. 1d. The carbon peak that appeared at 284.8 eV was due to the hydrocarbon originating from the XPS instrument itself used as the standard. The C 1s spectrum (Fig. 1e) could be deconvoluted into three peaks centred at 284.7, 288.2, and 293.4 eV, corresponding to C=C/C–C, C–N and C–F, respectively. The C=C and C–N were derived from the C_3_N_4_ in Mn_3_O_4_/C_3_N_4_-JMC, while C–F was derived from the carbonized pollen. The three peaks of N 1s at 398.8, 400.5, and 404.6 eV (Fig. 1f) could be attributed to C=N–C, C–N–H, and O–N. The Mn 2p peaks (Fig. 1g) located at 642.2 and 653.3 eV were fitted to Mn 2p_3/2_ Mn 2p_1/2_, respectively. Moreover, the O 1s spectra showed three components (Fig. 1h), with the C–O–Mn bond (532.2 eV) for Mn_3_O_4_/C_3_N_4_-JMC revealing that some of the O of Mn_3_O_4_ was involved in bonding with the C of C_3_N_4_.

Fig. S1† shows the DTA curves of Mn_3_O_4_/C_3_N_4_-JMC, pure C_3_N_4_, and Mn_3_O_4_. The small and linear loss of weight below 100 °C was due to the evaporation of water. Compared to the pure C_3_N_4_ and Mn_3_O_4_ samples, the weight of Mn_3_O_4_/C_3_N_4_-JMC showed a slight decrease from 120 °C to 500 °C, while the DTA curve displayed a broad exothermic peak, due to the degradation of carbonized sunflower pollen (C) and the partial decomposition of C_3_N_4_ in the composites. Moreover, the DTA curve of the pure C_3_N_4_ samples also indicated that C_3_N_4_ was partially pyrolyzed in this temperature range. The next mass loss stage of Mn_3_O_4_/C_3_N_4_-JMC between 500 °C and 700 °C could be ascribed to the decomposition of C_3_N_4_, along with an exothermic reaction, as shown in the DTA curve. The same result was also reflected in the DTA curve of pure C_3_N_4_. Compared with the relevant literature,^42^ the exothermic peak of C_3_N_4_ was shifted towards a lower temperature. It can be inferred from this that the formation of the Mn_3_O_4_/C_3_N_4_ heterojunction affected the temperature resistance of Mn_3_O_4_/C_3_N_4_-JMC.^43^

**Fig. 2:**
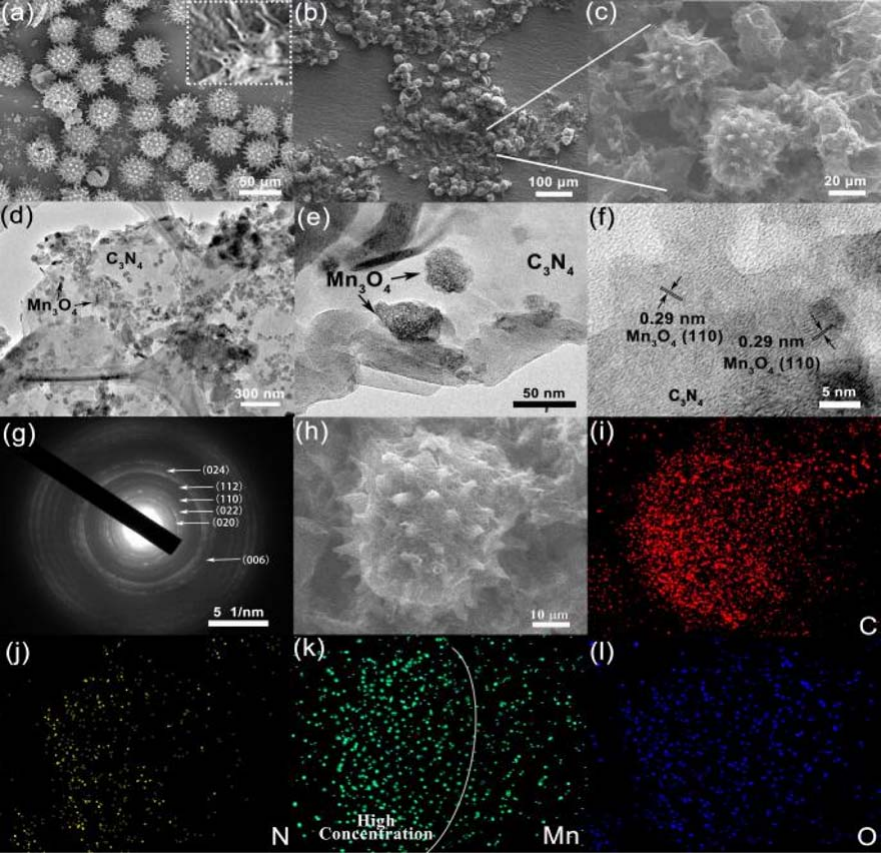
FESEM images of sunflower pollens (a) and Mn_3_O_4_/C_3_N_4_-JMC (b and c). TEM images (d–f), selected area electron diffraction (g) of Mn_3_O_4_/C_3_N_4_-JMC. Energy-dispersive X-ray mappings of Mn_3_O_4_/C_3_N_4_-JMC (h–l).

The FESEM and TEM images further revealed the micro- and nanostructure of the obtained samples. As shown in Fig. 2a, the sunflower pollen had a hollow and spiked structure with a smooth surface and a uniform diameter of around 30 μm. It is worth noting that the needle structure offers a large surface area for the subsequent synthesis of C_3_N_4_, while the hollow structure reduces the weight of the micromotor catalyst and contributes to fast motion. The FESEM image of Mn_3_O_4_/C_3_N_4_-JMC showed that free-standing C_3_N_4_ nanosheets were grown uniformly on the surface of the sunflower pollen in an edge-to-face stacking mode to form a 3D hierarchical structure, avoiding the agglomeration of C_3_N_4_ sheets (Fig. 2b and c). Furthermore, the TEM images further revealed the detailed structural characteristics of Mn_3_O_4_/C_3_N_4_-JMC. Irregular spherical Mn_3_O_4_ particles with an average grain size of 30 nm were uniformly grown on the surface of the interlaced C_3_N_4_ nanosheets (Fig. 2d and e). As shown in Fig. 2f, there was a lattice fringe of 0.29 nm ascribed to the (110) plane of Mn_3_O_4_. Besides, Fig. 2g presents the SAED photograph of Mn_3_O_4_/C_3_N_4_-JMC, revealing clear diffraction rings corresponding to the (020), (022), (110), and (112) crystal planes of Mn_3_O_4_, consistent with the XRD characterization results. Fig. 2h–l show the energy-dispersive X-ray (EDX) mappings of the Mn_3_O_4_/C_3_N_4_-JMC. The elements C, N, C, Mn, and O were well distributed on the surface of the catalyst, but the distribution of Mn was more focused on one side of the microsphere (Fig. 2l), confirming the successful semi-coating of Mn_3_O_4_ on the surface of the catalyst.

### Motion behaviours of the Mn_3_O_4_/C_3_N_4_-JMC micromotor catalyst

**Fig. 3:**
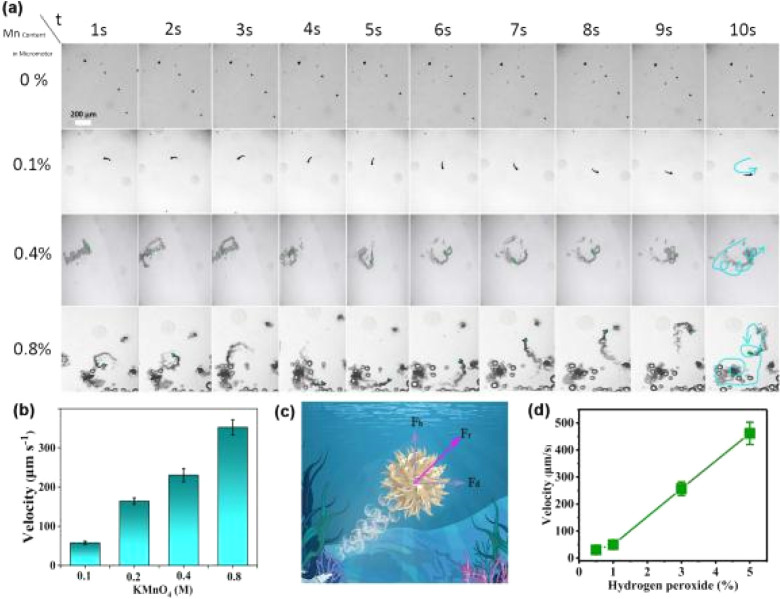
Time-lapse images of Mn_3_O_4_/C_3_N_4_-JMC in 1 wt% H_2_O_2_ solution containing 0.5 wt% sodium dodecyl sulfonate under different Mn contents (a). Bar chart of micromotor catalyst velocity under different Mn contents (b). The force condition of the micromotor catalyst during movement (c). Effect of fuel H_2_O_2_ concentration on the movement rate (d).

The self-propelled movement of Mn_3_O_4_/C_3_N_4_-JMC was powered by the O_2_ bubbles generated from the H_2_O_2_ decomposition by Mn_3_O_4_. Video S1† displays the movement of Mn_3_O_4_/C_3_N_4_-JMC in different concentrations of H_2_O_2_ solution containing 0.5% sodium dodecyl sulfonate. No regular curve or circular motion were observed in Video S1.† Moreover, the amount of manganese precursor had an important effect on the motor power. The effect of KMnO_4_ concentration on the velocity of Mn_3_O_4_/C_3_N_4_-JMC is shown in Fig. 3a. The average velocity increased from 57.83 ± 2.24 μm s^−1^ to 352.45 ± 19.23 μm s^−1^ as the KMnO_4_ concentration was increased from 0.1 to 0.8 M (Fig. 3b). In addition, the hollow structure of the catalyst generated buoyancy in the fluid, which also had a positive effect on the rapid movement of the catalyst. As shown in Fig. 3c, the resultant force (*F*_r_) for motion was a combination of the driving force (*F*_d_) produced by the bubbles and the buoyancy (*F*_b_) of the motor itself. The O_2_ bubbles generated a strong momentum that propelled the catalyst forward with a velocity of 30.4 ± 3.4, 49.6 ± 7.2, 256.8 ± 25.2, 462.6 ± 41.2 μm s^−1^ in 0.5, 1, 3, and 5 wt% H_2_O_2_ (Fig. 3d), respectively. Besides, the drag force, mechanical work, chemical input power, and working efficiency are summarized in Table 1, where it can be observed that as the concentration of hydrogen peroxide increased, all four parameters gradually increased. The work efficiency of the micromotor catalyst was in the order of 10^−7^. These excellent kinetic and mechanical parameters lay a foundation for the application of such catalysts in micro-space or trace concentration fields.

**Table 1:** Velocity and work efficiency of Mn_3_O_4_/C_3_N_4_-JMC in different concentrations of H_2_O_2_

H_2_O_2_/%	*ν*/10^−6^ m s^−1^	*F*_drag_/10^−9^ N	*P*_mecha_/10^−15^ W per motor	*P*_chem_/10^−8^ W per motor	*η*/10^−7^
0.5	30.4	1.79	489.7	45.6	5.67
1	49.6	2.79	623.01	62.8	9.92
3	256.8	6.95	2445.01	83.1	29.42
5	462.6	20.06	11 991.87	314.7	38.11

### Micromotor-assisted degradation of CBZ

**Fig. 4:**
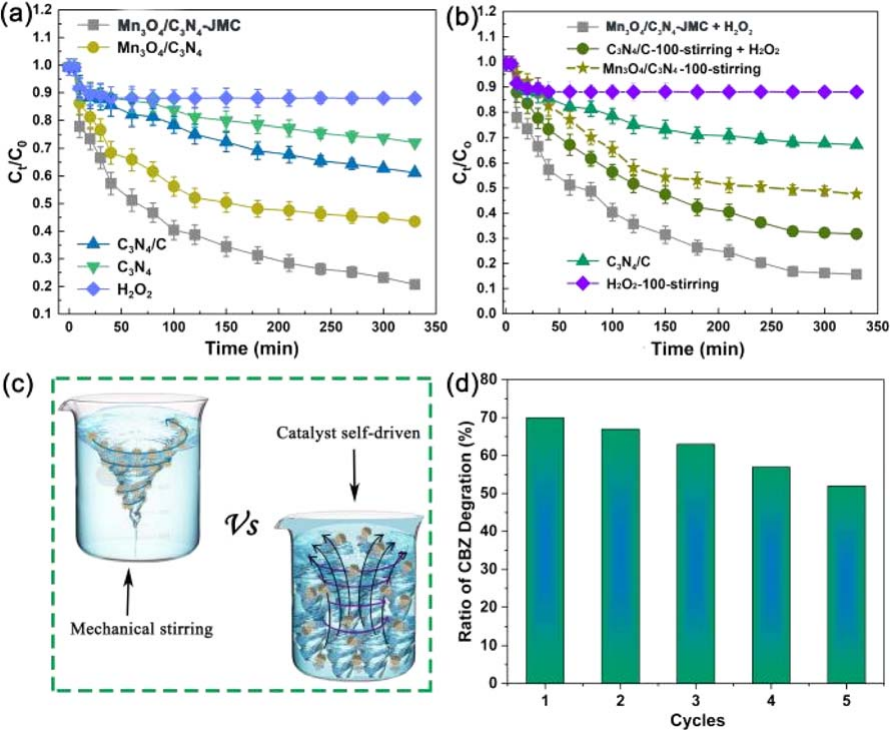
Degradation of CBZ using different samples in 3% mass fraction of H_2_O_2_ (a). CBZ degradation by Mn_3_O_4_/C_3_N_4_-JMC, C_3_N_4_/C-100-stirring (mechanical stirring at a speed of 100 rpm with H_2_O_2_), Mn_3_O_4_/C_3_N_4_-100-stirring (mechanical stirring at a speed of 100 rpm without H_2_O_2_), C_3_N_4_/C without H_2_O_2_ and with H_2_O_2_ with mechanical stirring at 100 rpm (b) schematic of the CBZ degradation principle *via* mechanical agitation and micromotor catalyst motion (c); reusability of the catalyst for CBZ degradation (d).

Fig. 4a shows the CBZ degradation by different samples. About 79.3% and 55.5% CBZ were degraded by Mn_3_O_4_/C_3_N_4_-JMC and Mn_3_O_4_/C_3_N_4_ in 300 min, which were much higher rates than those achieved by g-C_3_N_4_, C_3_N_4_/C, and H_2_O_2_. The degradation rate of Mn_3_O_4_/C_3_N_4_-JMC was always higher than that of Mn_3_O_4_/C_3_N_4_, especially in the degradation stage at low CBZ concentration, which was due to the active contact of the catalyst with the pollutant molecules. Besides, several control experiments were carried out to verify the roles of movement in the degradation of CBZ. Fig. 4b shows CBZ degradation by under different conditions: Mn_3_O_4_/C_3_N_4_-JMC, C_3_N_4_/C-100-stirring (mechanical stirring with speed of 100 rpm with H_2_O_2_), Mn_3_O_4_/C_3_N_4_-100-stirring (mechanical stirring with speed of 100 rpm without H_2_O_2_), C_3_N_4_/C without H_2_O_2_ and H_2_O_2_ with mechanical stirring 100 rpm. The comparison results showed that the Mn_3_O_4_/C_3_N_4_-JMC exhibited higher catalytic activity towards CBZ than static C_3_N_4_/C and C_3_N_4_/C-100 under mechanical stirring with a speed of 100 rpm, indicating the positive contribution of autonomous movement. It is an important point that the random autonomous movement of the catalyst results in turbulence of the reactive liquid, facilitating more efficient contact between the active sites and the contaminants. Conventional mechanical agitation results in liquid flow in a certain direction, and the active site is in contact with contaminants only to a certain extent (Fig. 4c). Therefore, the degradation of pollutants by our catalyst system is superior to mechanical agitation in a certain range. Meanwhile, the reusability of the catalyst for the catalytic degradation of CBZ was studied. As shown in Fig. 4d, the CBZ removal rate was 54.7% at the end of the 5th cycle in 100 min, slightly lower than that for the first cycle (69.3%), indicating its high reusability for CBZ degradation. Besides, to clarify the active species responsible for the catalysis activity, and as shown in Fig. S2,† IPA, EDTA, and *P*-benzoquinone were used as scavengers for ˙OH, oxygen vacancies, and ˙O^2−^.^44^ It was found that hydroxyl free radicals (˙OH) and superoxide free radicals (˙O^2−^) were produced in large quantities in the process of degradation. Comparatively, ˙OH radicals were the main active species.

The kinetic study for the photocatalytic degradation of CBZ was investigated using pseudo-first-order and pseudo-second-order kinetic models.^45,46^Fig. 4a presents the photocatalytic degradation plot of CBZ using Mn_3_O_4_/C_3_N_4_-JMC, in which the operating condition of 50 mg per L photocatalyst loading was applied. The pseudo-first-order kinetics based on Langmuir–Hinshelwood kinetics when a small initial concentration of the reactant is used can be described by eqn (2):

2 −ln*C*_*t*_/*C*_0_ = *k*_1_*t*

where *C*_0_ (mg L^−1^) and *C*_*t*_ (mg L^−1^) are CBZ concentrations at initial and reaction times, respectively, *t* is the irradiation time, and *k*_1_ is the apparent first-order rate constant of CBZ degradation. Fig. S3(a)† depicts the plot of −ln*C*_*t*_/*C*_0_*vs.* time for Mn_3_O_4_/C_3_N_4_-JMC within 100 min irradiation. The slope of the linear fitted plot depicts *k*_1_, which was calculated as 0.0088 min^−1^ and the coefficient of determination (*R*^2^) was obtained as 0.9253. Therefore, the pseudo-first-order kinetics model showed an unsatisfactory quality of linear fitting.

The pseudo-second-order kinetic model can be described by eqn (3):

3 1/*C*_*t*_ − 1/*C*_0_ = *k*_2_*t*

where *k*_2_ (L mg^−1^ min^−1^) is the second-order kinetics rate constant and was determined from a linear fitting of the data. Fig. S3(b)† shows the plot of (1/*C*_*t*_–1/*C*_0_) *vs. t* within 300 min irradiation. From the slope of the linear fitted plot, *k*_2_ could be calculated as 0.0144 L mg^−1^ min^−1^ and *R*^2^ was obtained as 0.9793. A much higher fitting quality was obtained by the pseudo-second-order kinetics model equation. Based on these results, it could be concluded that the photodegradation of CBZ using Mn_3_O_4_/C_3_N_4_-JMC followed a pseudo-second-order reaction, indicating that the rate of reaction with the autonomous movement catalyst was not only related to the concentration of reactants, but also to the concentration of the intermediate or transformation products generated by the reaction.

### Stability of the Mn_3_O_4_/C_3_N_4_-JMC micromotor catalyst

**Fig. 5:**
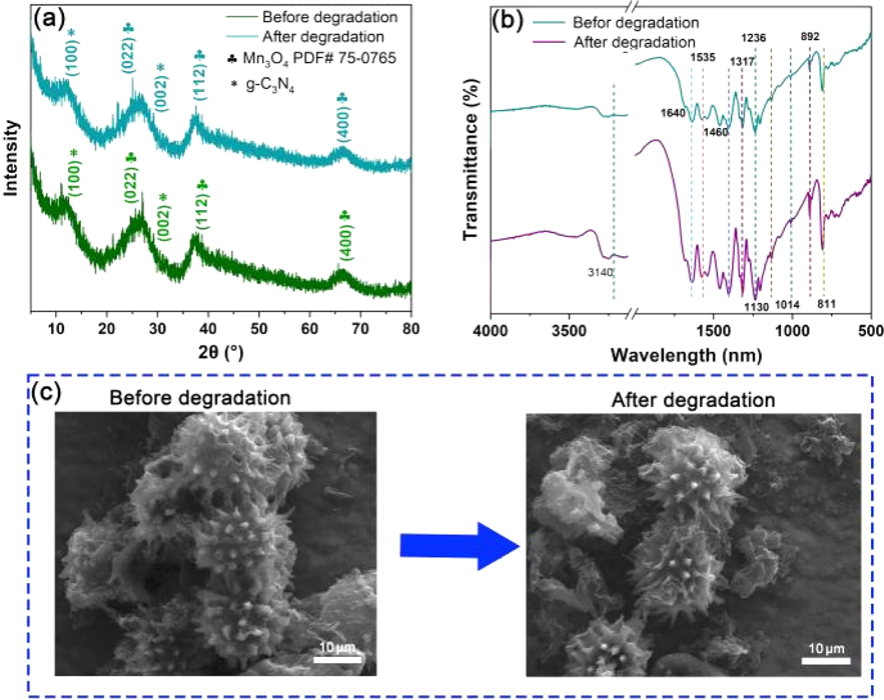
XRD patterns (a) and FT-IR spectra (b) before and after CBZ degradation by Mn_3_O_4_/C_3_N_4_-JMC. FESEM images of Mn_3_O_4_/C_3_N_4_-JMC before and after CBZ degradation (c).

Based on the degradation efficiency of CBZ, the stability of Mn_3_O_4_/C_3_N_4_-JMC was tested under the same conditions, as shown in Fig. 5a and b. Comparing the XRD and FT-IR patterns before and after the degradation of CBZ, there was no significant change in the phase composition and bonding state in the Mn_3_O_4_/C_3_N_4_-JMC samples, indicating the high chemical stability and reusability of the catalysts in the process of CBZ degradation. Furthermore, the FESEM image of Mn_3_O_4_/C_3_N_4_-JMC before and after five cycles (Fig. 5c) shows that its morphology remained almost unchanged after the cyclic testing, demonstrating its high stability and reusability.

### Energy band structure analysis of Mn_3_O_4_/C_3_N_4_-JMC by electrochemical studies and DFT theory

**Fig. 6:**
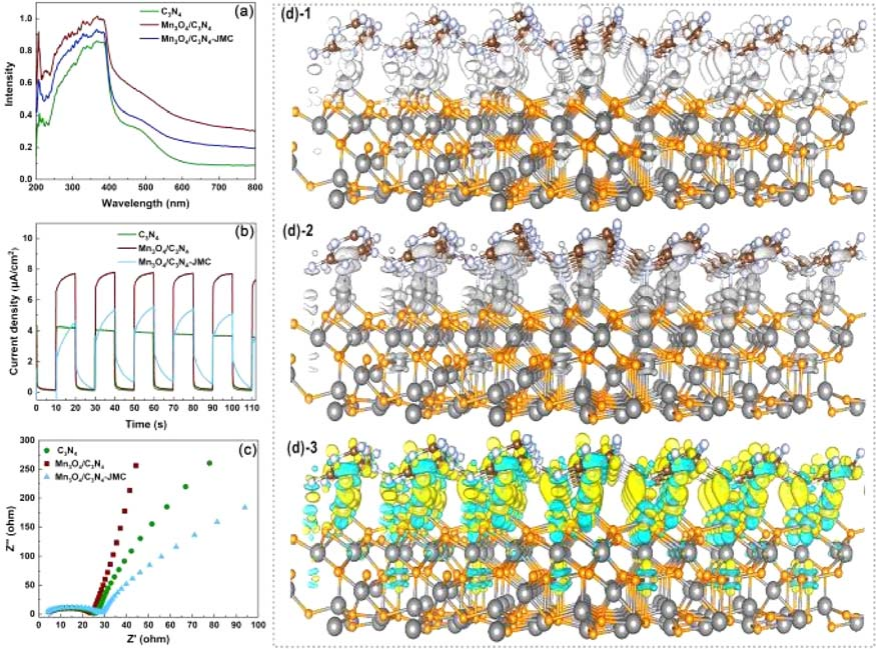
UV-visible diffuse reflectance spectra of the as-prepared C_3_N_4_, Mn_3_O_4_/C_3_N_4_, and Mn_3_O_4_/C_3_N_4_-JMC samples (a). Transient photocurrent density of the samples (b). Electrochemical impedance spectra of C_3_N_4_, Mn_3_O_4_/C_3_N_4_, and Mn_3_O_4_/C_3_N_4_-JMC (c). Charge density difference between Mn_3_O_4_ and g-C_3_N_4_ interface. Positive part (d)-1, negative part (d)-2, and mixed distribution (d)-3. Yellow stands for the positive part, which indicates charge accumulation, whereas light green indicates charge depletion after hetero-contact formation. Up: g-C_3_N_4_, down: Mn_3_O_4_.

To reveal the degradation mechanism of carbamazepine, light-response, photocurrent, and electrochemical impedance tests were carried out to evaluate the photocatalytic activity of the catalyst. As shown in Fig. 6a, Mn_3_O_4_/C_3_N_4_ exhibited a shift towards a long wavelength region compared to C_3_N_4_, indicating that Mn_3_O_4_/C_3_N_4_ had a higher adsorption ability for visible light than C_3_N_4_; while Mn_3_O_4_/C_3_N_4_-JMC showed a higher adsorption ability of C_3_N_4_, due to the carbonization of pollen (C), which increased the separation of photoelectrons. Meanwhile, Mn_3_O_4_/C_3_N_4_-JMC had a higher photocurrent intensity than g-C_3_N_4_ (Fig. 6b), suggesting the stronger charge-transfer ability of Mn_3_O_4_/C_3_N_4_-JMC than g-C_3_N_4_. Besides, the electrochemical analysis showed that Mn_3_O_4_/C_3_N_4_-JMC displayed a smaller hemicycle radius than Mn_3_O_4_/C_3_N_4_ and g-C_3_N_4_ in the electrochemical impedance spectroscopy analysis (Fig. 6c), indicating a more efficient production of photoexcited electrons and holes.

To further understand the charge-transport effect between g-C_3_N_4_ and Mn_3_O_4_, DFT calculations were performed to illustrate the charge-transfer property. Fig. 6(d)-1 and (d)-2 show the charge-transfer situation in the interface of g-C_3_N_4_ and Mn_3_O_4_. Moreover, Fig. 6(d)-3 exhibits the charge-transfer situation in the interface of the heterojunction g-C_3_N_4_/Mn_3_O_4_. It can be clearly observed from Fig. 6(d)-3 that there was obvious charge transfer between the two phases, verifying the hetero-effect that the loading of Mn_3_O_4_ could affect the electronic structure on the surface of the 2D g-C_3_N_4_.^47–49^

**Fig. 7:**
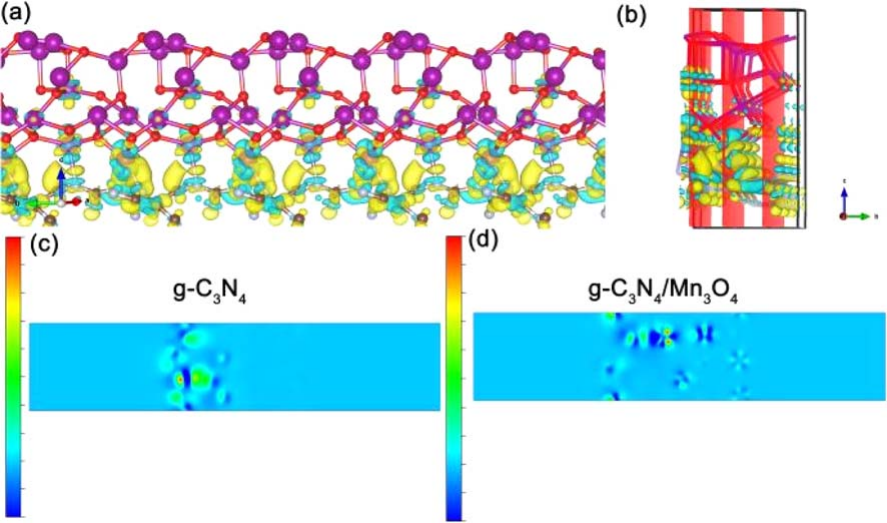
Charge difference distribution (a) (b) and electronic location function analysis of the g-C_3_N_4_ (c) and g-C_3_N_4_/Mn_3_O_4_ composite (d).

Fig. 7(a)–(d) exhibit the charge difference distribution and the corresponding electronic location function images to reveal the charge separation condition between Mn_3_O_4_ and g-C_3_N_4_. It is obvious that when the heterostructure between the g-C_3_N_4_ and Mn_3_O_4_ was formed, the cyan and yellow region show that there was an accumulation of electrons migrating from g-C_3_N_4_ to the Mn_3_O_4_. Meanwhile, the electronic location function image demonstrated that there existed a distinct covalent interaction between the C atom of g-C_3_N_4_ triazine and the Mn layer, forming a charge-transfer channel for transfer from g-C_3_N_4_ to the Mn_3_O_4_ nanosheets.^50–52^ So the above experimental test and theoretical calculation together confirmed that when g-C_3_N_4_ and Mn_3_O_4_ formed a composite, the charge separation and transfer ability between them could be dramatically improved compared to that of their counterparts alone.

**Fig. 8:**
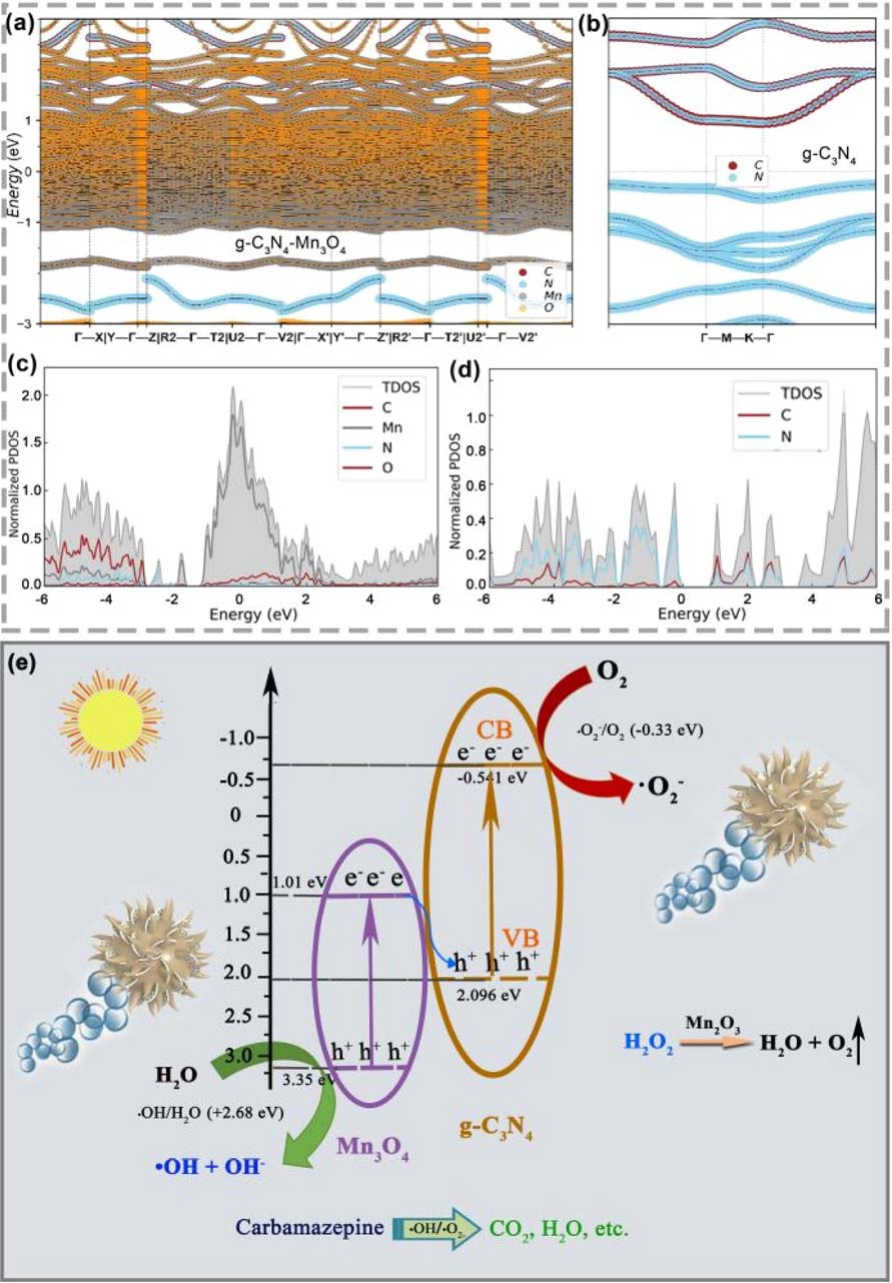
Projected-to-element band structures of Mn_3_O_4_/C_3_N_4_ (a) and g-C_3_N_4_ (b). Projected-to-element density of states (DOS) of Mn_3_O_4_/C_3_N_4_ (c) and g-C_3_N_4_ (d). Schematic of the photogenerated electron and hole migration and separation on the Mn_3_O_4_/C_3_N_4_-JMC heterojunction under visible-light irradiation (e).

The results and analysis in Fig. 5(a)–(c) prove that the heterojunction structure can significantly improve the electron conduction efficiency. Likewise, the band structure and density of states calculation were conducted to confirm the results. Fig. 8 shows the band structure of the heterostructure and density of states results of g-C_3_N_4_/Mn_3_O_4_ and g-C_3_N_4_. It is worth noting that the band gap was underestimated by the functional PBE, but it made the comparison in the identity accuracy level. As shown in Fig. 8a, the O and Mn element bands occupied the dominant Fermi level and overlapped the C and N elements, indicating the hetero-effect charge transfer and sharing between the two different phases. In terms of the band structure (Fig. 8b), it could be observed that the pristine g-C_3_N_4_ had a clear band gap, while the band gap disappeared in the heterogeneous structure. Furthermore, intensive crossing to the Fermi level (0 eV calibrated) indicated the better electronic conductivity. Moreover, the projected-to-element density of states was investigated and the results analyzed. From Fig. 8c, it could be observed that the most prominent peak occupied the Fermi level while isolated g-C_3_N_4_ was not found, in line with the experimental observation and previous band calculations, which confirmed the better electronic conductivity of the hetero-interface of g-C_3_N_4_/Mn_3_O_4_ (Fig. 8d). Therefore, constructing a multi-interface is a critical step for g-C_3_N_4_-based materials. To explain the mechanism of photocatalysis, the valence band (VB) and conduction band (CB) edges of g-C_3_N_4_ and Mn_3_O_4_ nanosheets were estimated. The CB edge values were calculated to be 1.01 and −0.541 eV, while the VB edge values were 3.35 and 2.096 eV for Mn_3_O_4_ and g-C_3_N_4_, respectively.^53^ According to the above results, both g-C_3_N_4_ and Mn_3_O_4_ nanosheets could generate electrons and holes in the heterojunction under visible-light irradiation (Fig. 8e). The result shows that the CB edge of Mn_3_O_4_ was lower than the redox potential of O_2_/˙O_2_^−^ (−0.28 eV), which means the electrons in the CB of Mn_3_O_4_ could not form ˙O_2_^−^ radicals.^54^ Similarly, since the VB edge of g-C_3_N_4_ was higher than the redox potential of ˙OH/OH^−^ (2.68 eV), the holes left in the VB of g-C_3_N_4_ could not form ˙OH radicals.^55,56^ However, the photocatalytic degradation performance also decreased after adding the hole-trapping agent (Fig. S2†), indicating that both electrons and holes were involved in photocatalytic degradation. Therefore, the g-C_3_N_4_/Mn_3_O_4_ nanosheets should form a Z-scheme heterojunction.^57–59^ When exposed to visible light, the electrons transit from the VB of g-C_3_N_4_ and Mn_3_O_4_ to CB, leaving holes in the VB. The photogenerated electrons in the CB of Mn_3_O_4_ transfer to the VB of g-C_3_N_4_. Therefore, holes are left in the VB of Mn_3_O_4_, while electrons are left in the CB of g-C_3_N_4_. The holes and electrons will react with OH^−^ and O_2_ to form free radicals with strong oxidation and deoxidization, as confirmed by the experimental capture of active species, which further oxidize CBZ molecules.

In addition, it is worth mentioning that, as an artificial active object, the micromotor catalyst can enhance mass transfer in the solution and improve the interaction between the active surface and the target pollutants. Hence, combined with the degradation path and assisted by the self-propelled motion of the catalyst, a very high CBZ degradation rate was obtained.

## Conclusions

In summary, an urchin-like Mn_3_O_4_/C_3_N_4_ Janus micromotor catalyst was precisely designed and synthesized for the dynamic photocatalytic degradation of carbamazepine from sewage. The asymmetric distribution of Mn_3_O_4_ on the surface of the catalyst was achieved by a facile semi-coating method. The self-propulsion of the Janus micromotor catalyst was achieved with a speed of 223.5 ± 7.0 μm s^−1^ through the O_2_ bubbles generated from the decomposition of H_2_O_2_ by Mn_3_O_4_. Furthermore, the photoelectrons generated from the Mn_3_O_4_/C_3_N_4_ heterojunction could create active species and promote the photocatalytic degradation activity of the catalyst under simulated sunlight irradiation. Consequently, nearly 70% of CBZ could be degraded within 5 h with the help of the movement. Besides, density functional theory calculations proved that the formation of the heterojunction structure promoted the generation of photogenerated carriers. Therefore, these features endow the micromotor catalyst with exciting potential in environmental remediation fields.

## Data availability

The authors confirm that the data supporting the findings of this study are available within the article and its ESI.†

## Author contributions

Jie Yang: conceptualization; data curation; formal analysis; investigation; visualization; writing – original draft. Wenning Yang: data curation; formal analysis. Chao Zhang: conceptualization; formal analysis. Jian Gong: investigation; supervision. Jia Li: conceptualization; funding acquisition; supervision; writing – review & editing; Ming Xu: conceptualization; resources; funding acquisition. Chengzhang Liu: data curation, resources.

## Conflicts of interest

There are no conflicts to declare.

## Supplementary Material

RA-014-D4RA04980B-s001

RA-014-D4RA04980B-s002
