# Supplementary material for: Synergistic self-driven and heterogeneous effect of a biomass-derived urchin-like Mn3O4/C3N4 Janus micromotor catalyst for efficient degradation of carbamazepine
Source: RSC Adv. 2024 Sep 12;14(39):28904–14. doi: 10.1039/d4ra04980b (PMC11391418; doi:10.1039/d4ra04980b)
Supplement: RA-014-D4RA04980B-s001 [file RA-014-D4RA04980B-s001.pdf]

Supplementary Information (SI) for RSC Advances.
This journal is © The Royal Society of Chemistry 2024

Electronic Supplementary Information

Synergy of Self-Driven and Heterogeneous Effect on Biomass-Derived Urchin-like Mn_3O_4/C_3N_4 Janus Micromotor Catalyst for Efficient Degradation of Carbamazepine

Jie Yang,[1] Wenning Yang,[2#] Chao Zhang,[3] Jian Gong,[1] Ming Xu,[4] Jia Li,[5] Chengzhang Liu [5]*

[1] Department of Pharmaceutical and Bioengineering, ZiBo Vocational Institute, ZiBo, 255000, P. R. China

[2] Shandong Provincial Key Laboratory of Chemical Energy Storage and Novel Cell Technology, and School of Chemistry and Chemical Engineering, Liaocheng University, Liaocheng 252000, P. R. China

[3] School of Artificial Intelligence and Big Data, ZiBo Vocational Institute, ZiBo, 255000, P. R. China

[4] State Key Laboratory of Chemical Resource Engineering, Beijing University of Chemical Technology, Beijing, 100029, P. R. China

[5] School of Material Science and Engineering, University of Jinan, Jinan, 250022, China

[#] The authors contribute equally: Wenning Yang

** Corresponding author: mse_lij@ujn.edu.cn (Jia Li), +86139531854*

Keywords: Biomass, Heterostructure, Photocatalysis, Micromotor, Carbamazepine

Table of Content

Section 1: Supporting Video

Video S1 Motion behavior of $Mn_3O_4/C_3N_4/C$ Janus micromotor catalyst (Mn_3O_4/C_3N_4-JMC) in 1%, 3% and 5% H_2O_2 aqueous containing 0.5% of sodium dodecyl sulfonate.

Section 2: Figures

Figure S1 DSC curves of Mn_3O_4/C_3N_4-JMC.

Figure S2 Effect of active substance trapping agent on degradation.

Section 3: Tables

Table S1 Pore texture parameters of the obtained samples.

Section 4: The study of drag force, resistance and working efficiency of micromotor catalyst.

Section 2: Figures

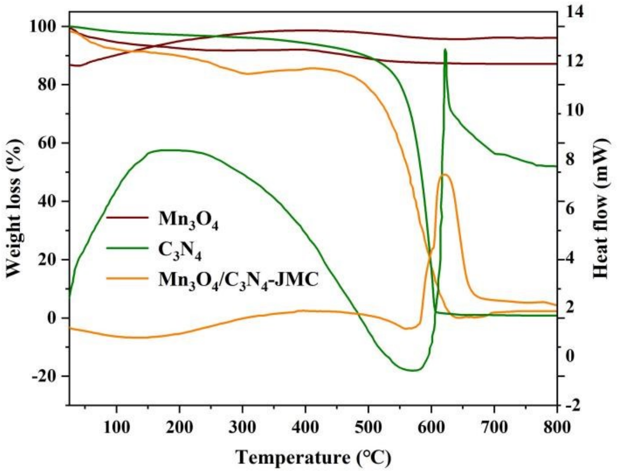

Figure S1 DTA curves of Mn_3O_4/C_3N_4-JMC, pure Mn_3O_4 and C_3N_4.

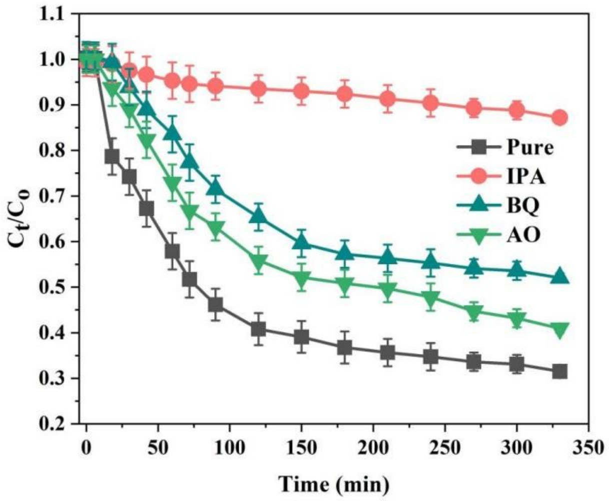

Figure S2 Effect of active substance trapping agent on degradation

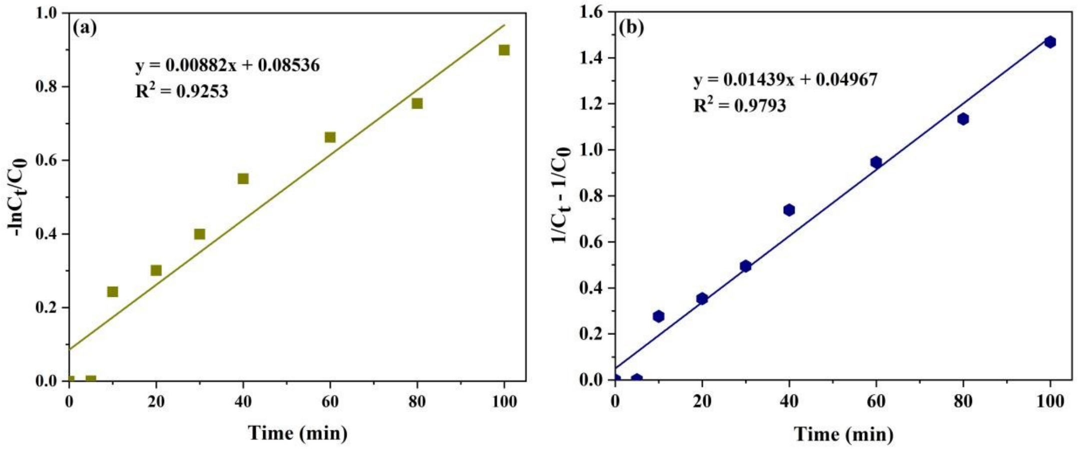

Figure S3 Plot of pseudo-first-order kinetics (a) and pseudo-second-order kinetics (b) for photocatalytic degradation of CBZ by Mn_3O_4/C_3N_4-JMC

Section 3: Tables

Table S1 Pore texture parameters of the obtained samples

Sample	S_{BET} ($m^2 \cdot g^{-1}$)	V_{Total} ($cm^3 \cdot g^{-1}$)	D_p (nm)
1#	92.81	0.3288	7.1
2#	98.63	0.4558	9.2
3#	81.35	0.2825	6.3

[a] S_{BET}: BET specific surface area; S_{Micro}: micropore surface area; V_{Total}: total pore volume; V_{Micro}: micropore volume; D_p: average pore

Section 4: The study of drag force, resistance and working efficiency of micromotor catalyst

The drag force, resistance and working efficiency of the Mn_3O_4/C_3N_4-JMC were studied. It is assumed that the drag force generated by the bubble breaking away from the catalyst surface is equal to the steady-state fluid power. Therefore, the speed and force of the catalyst under low Reynolds coefficient can be estimated by the following formula:

$$F_{drag} = 6\pi\mu\gamma\nu \tag{1}$$

ν($m \cdot s^{-1}$) is the speed of micromotor catalyst motion, μ(mPa·s) is fluid viscosity, and γ(m) is distance traveled in a given amount of time.

The kinetic energy of the micromotor catalyst driven by bubbles is actually converted from the chemical energy generated by the decomposition of H_2O_2 by Mn_3O_4. Therefore, the efficiency of the catalyst can be calculated by the following formula [1]:

$$\eta = P_{mecha}/P_{chem} \tag{2}$$

$$P_{mecha} = F_{drag} \cdot \nu = 6\pi\mu\gamma\nu^2 \tag{3}$$

$$P_{chem} = n \cdot \Delta r^{\theta} G \tag{4}$$

$$n = PV/RT \tag{5}$$

$$V = N \cdot 4\pi R_0^3/3 \tag{6}$$

η is the efficiency of the catalyst, P_{mecha} is catalyst mechanical power ($W \cdot motor^{-1}$), P_{chem} is chemical input power ($W \cdot motor^{-1}$), which is equal to the chemical energy of all the gas produced, V is the volume of an ideal gas. n is mol of gas (mol), T is thermodynamic temperature of an ideal gas (K), N is the number of bubbles per second, R_o is the diameter of bubble (m) [2].

Notes and references

(1) W. Gao, S. Sattayasamitsathit, J. Orozco, J. Wang, Highly efficient catalytic microengines: template electrosynthesis of polyaniline/platinum microtubes, J. Am. Chem. Soc. 133 (2011) 11862-11864.

(2) P. Kannan, T. Maiyalagan, E. Marsili, S. Ghosh, J. Niedziolka-Jonsson, M. Jonsson-Niedziolka, Hierarchical 3-dimensional nickel–iron nanosheet arrays on carbon fiber paper as a novel electrode for non-enzymatic glucose sensing, Nanoscale 8 (2016) 843-855.
